# Supplementary material for: Precise temporal regulation of alternative splicing during neural development
Source: Nat Commun. 2018 Jun 6;9:2189. doi: 10.1038/s41467-018-04559-0 (PMC5989265; doi:10.1038/s41467-018-04559-0)
Supplement: Supplementary file 1 — Supplementary Information [file 41467_2018_4559_MOESM1_ESM.pdf]

Supplementary Information Weyn-Vanhentenryck et al.

Precise temporal regulation of alternative splicing during neural development

Supplementary Discussion

Validation of developing cortex RNA-seq data quality and AS quantification.

To assess the quality of the mouse cortex RNA-seq data we generated, we initially examined expression of all annotated genes and splicing of all annotated cassette exons. Overall, biological replicates of the same age are highly correlated (Pearson correlation $r \geq 0.996$ for expression; $r \geq 0.991$ for splicing). In contrast, we observed dramatic changes in expression and alternative splicing between time points, particularly in the embryonic and neonatal cortices.

To further validate the results of splicing quantification, we performed semi-quantitative RT-PCR for 10 exons that were previously reported to have developmental splicing changes (Supplementary Dataset 3). Selection of these exons was independent of the RNA-seq data. Instead, the developmental splicing pattern of these exons reported in the literature was used to determine the time points we should include for RNA-seq profiling. One example of these exons is *Mapt* exon 10. This exon is almost completely skipped in the embryonic cortex, shows intermediate inclusion between P4 and P7, and reaches almost complete inclusion after P30 (Supplementary Fig. 1c). The observed pattern is consistent with previous observations[1]. Quantification of the exon based on the RNA-seq data and semi-quantitative RT-PCR gave very similar results (R^2=0.99; Supplementary Fig. 1d). High correlation between RNA-seq and PCR validation was also observed in the other tested exons (overall R^2=0.96; Supplementary Fig. 1e). These results confirm the reliability of the RNA-seq data and our quantification of exon inclusion levels.

Developmental splicing changes reflect pan-neuronal changes instead of changes in cell composition.

A recent study used RNA-seq to profile gene expression in all major cell types in the mouse cortex, including neurons, different types of glial cells and endothelial cells[12,13]. In a comparison of the splicing profiles of these cell types, we found that neurons show the most distinct splicing profiles and are the most similar to the whole cortex tissue. In addition, we did not observe global developmental splicing switches during the differentiation and maturation of oligodendrocytes that would explain the modules we identified (Supplementary Fig. 4a). Cortical neurons are broadly classified as either pyramidal neurons or interneurons, which differ in their abundance and developmental origins. To assess whether the different representation of pyramidal neurons and interneurons can bias the developmental splicing profiles we observed in cortex tissues, we compared splicing profiles of 19 glutamatergic neuronal subtypes and 23 GABAergic neuronal subtypes defined by single-cell RNA-seq of the primary visual cortex of the adult mice[2]. We observed no global differences between the two broad categories of neurons that are correlated with the modules we identified (Supplementary Fig. 4b).

Developmental origins of sensory receptor cells and sensory neurons.

The non-neuronal sensory receptor cells (enterochromaffin cells and sweet/bitter taste receptor cells) examined in this study derive from epithelial cells of non-neurogenic origin. OSNs and the somatic (from DRG and trigeminal ganglia) and visceral (from jugular and nodose ganglia) sensory ganglion neurons we analyzed arise from either the pre-placodal region or neural crest, which were derived from a zone of the ectoderm that borders the neural plate, or neural border[3]. For example, OSNs in the olfactory epithelium originate from cranial olfactory placode region developing from the neural plate border[4], the DRG sensory neurons develop from neural crest, and the trigeminal ganglion is thought to differentiate either from neural crest and the trigeminal placodes[5,6]. Importantly, the pre-placodal region and neural crest remain in close proximity to the neural plate and are subsequently coordinated throughout the development[7]. This common embryonic origin could provide one explanation for the distinct splicing program in the sensory neuron and ganglion neuron populations, as compared to CNS neurons, which derive from the neural plate. The rod and cone photoreceptors are derived from the optic vesicles that originate from the neural plate. They are distinct from the “canonical” CNS neurons in that the optic vesicles originate from the “outgrowth” of the developing brain and it interacts with the lens placode in close proximity, which could induce morphological and molecular changes in both tissues during retina development[8,9].

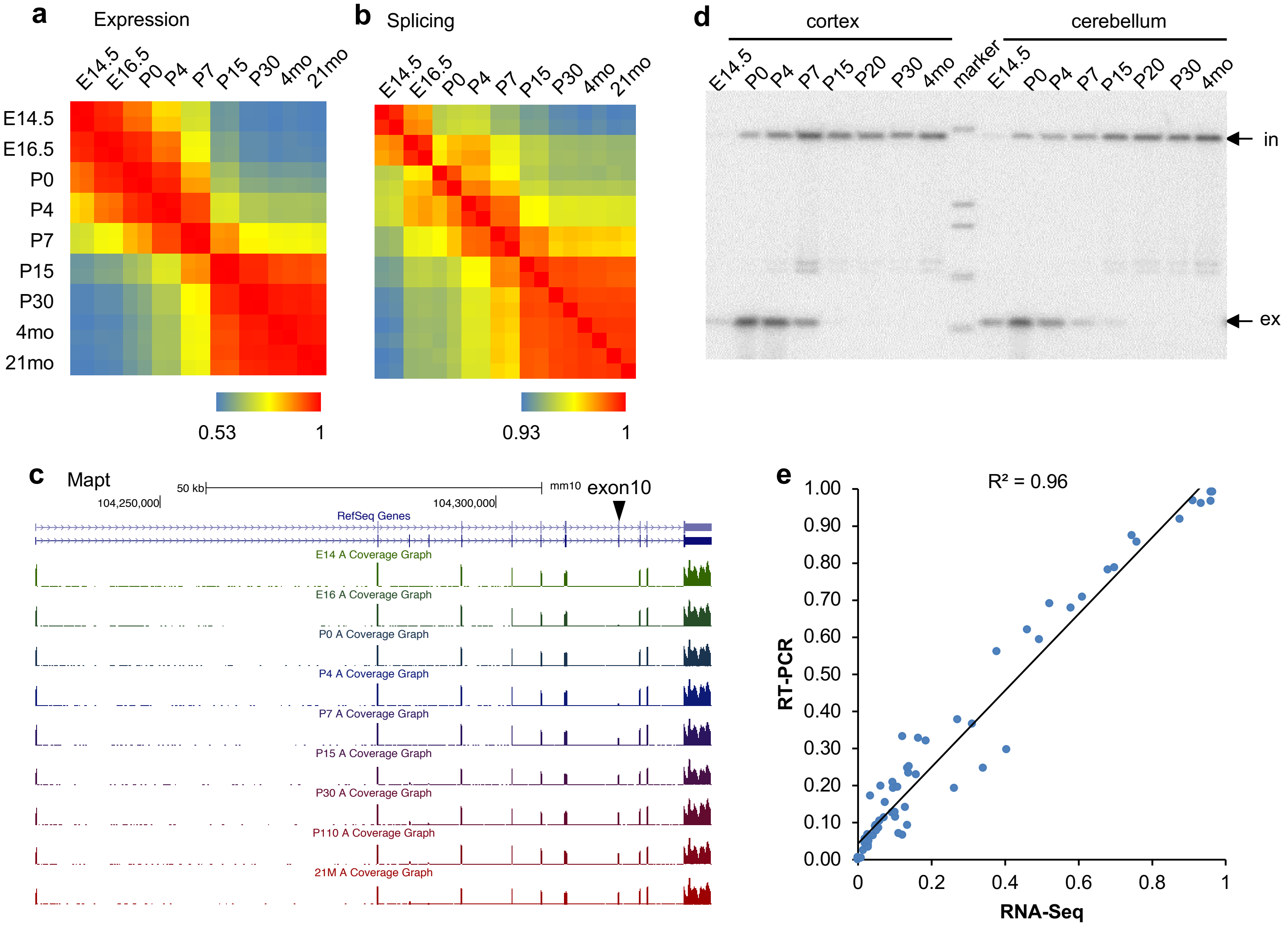

Supplementary Figure 1: Validation of developing cortex RNA-seq data and AS quantification. a, Pairwise correlation of gene expression in mouse cortices between different developmental stages. Duplicates were analyzed at each time point. **b**, Similar to (a), but pairwise correlation estimated from inclusion level of ~16,000 known cassette exons. **c**, *Mapt* exon 10 as an example of developmentally regulated exons. **d**, Semi-quantitative RT-PCR validation of splicing for *Mapt* exon 10 in the cortex and cerebellum. **e**, A summary of splicing quantification of 10 exons at different developmental stages using RT-PCR and RNA-seq.

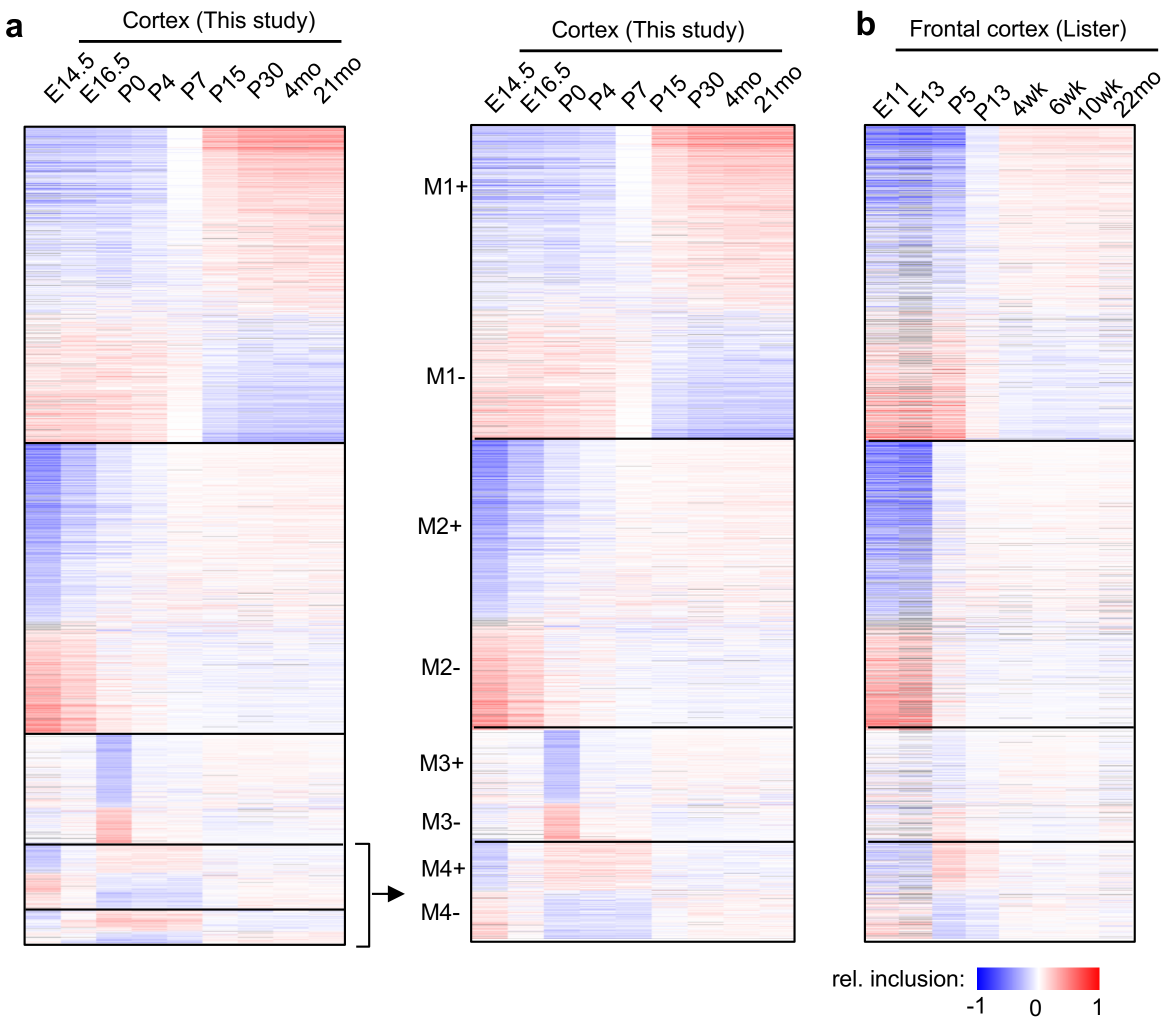

Supplementary Figure 2: Robustness of the modular organization of developmentally regulated alternative exons in an independent dataset. a, Related to **Fig. 1d** in the main text, WGCNA initially identified five modules. Modules 4 and 5, together composed of 353 of 2,883 exons, both capture non-monotonic splicing changes with similar temporal patterns and were merged manually into a final module M4 presented in the paper. **b**, Comparison of splicing in an independent dataset derived from mouse frontal cortices at different developmental stages from E11 to adult. Exons are shown in the same order as in the right panel in (**a**). Exons in three of the four modules (M1, M2, and M4) show very similar developmental switches. Since P0 was not included in this second dataset, the reproducibility of the distinct splicing pattern of exons in module M3 could not be evaluated here (however, this module is reproducible in other datasets that include P0 or P1, see e.g., Fig. 2a in the main text). Note that the denser sampling of time points before P15 in our cortex RNA-seq dataset allows us to capture more dynamics.

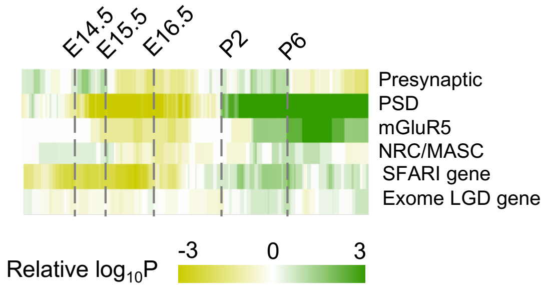

Supplementary Figure 3: Functional enrichment of genes with splicing switches with specific timing. Related to **Fig. 1e** in the main text, exons were ranked based on the timing of splicing switches. Exons in each sliding window (with a window size of 300 exons) were compared to all casette exons with sufficient read coverage in the cortex to evaluate enrichment of genes with specific functional annotations (see Methods).

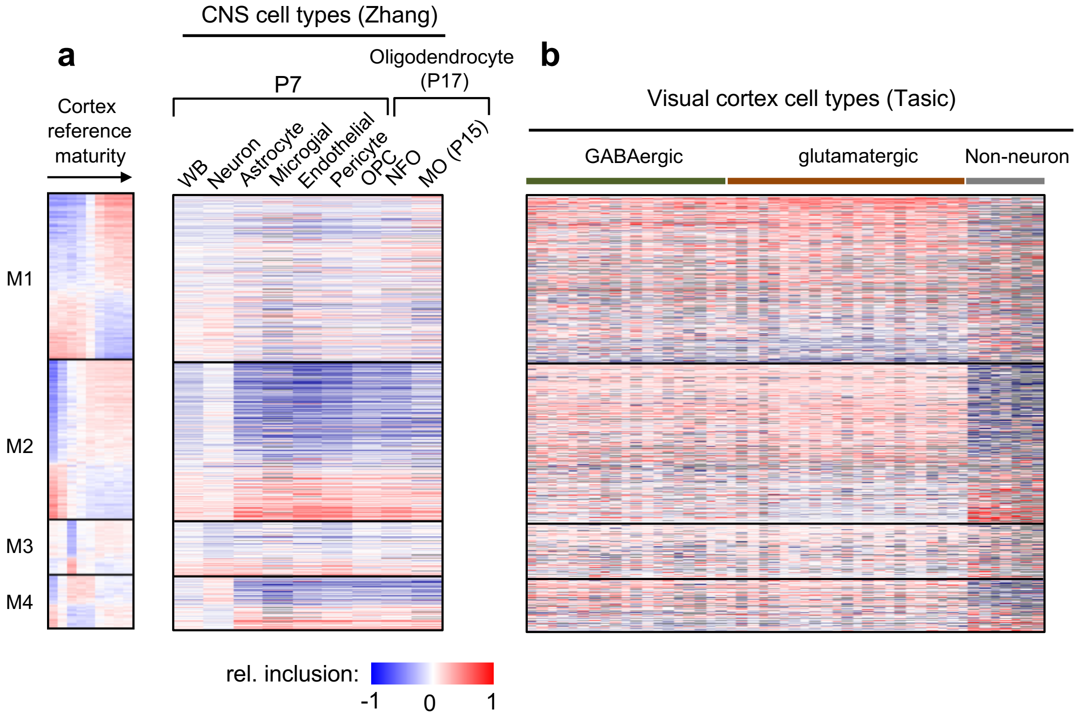

Supplementary Figure 4: The splicing profile of different cell types in the cortex. Related to **Fig. 2** in the main text, splicing profiles of module exons are shown for different cell types isolated from the cortex. In each panel, exons are shown in the same order as in the cortex refence on the left. **a**, In the Zhang et al. dataset, oligodendrocyte progenitor cells (OPCs), newly formed oligodendrocyte (NFO) and myelinated oligodendrocytes (MO) were purified from P17 mice, while the remaining samples were purified from P7 mice. **b**, The Tasic et al. dataset are single-cell RNA-seq profiles from the primary visual cortex of adult mice. In this dataset, splicing profiles were quantified by pulling reads from cells that are core members of each cell type. Specific subtypes were ordered based on the broad categories (GABAergic interneurons, glutamatergic pyramidal neurons, and non-neuronal cell types).

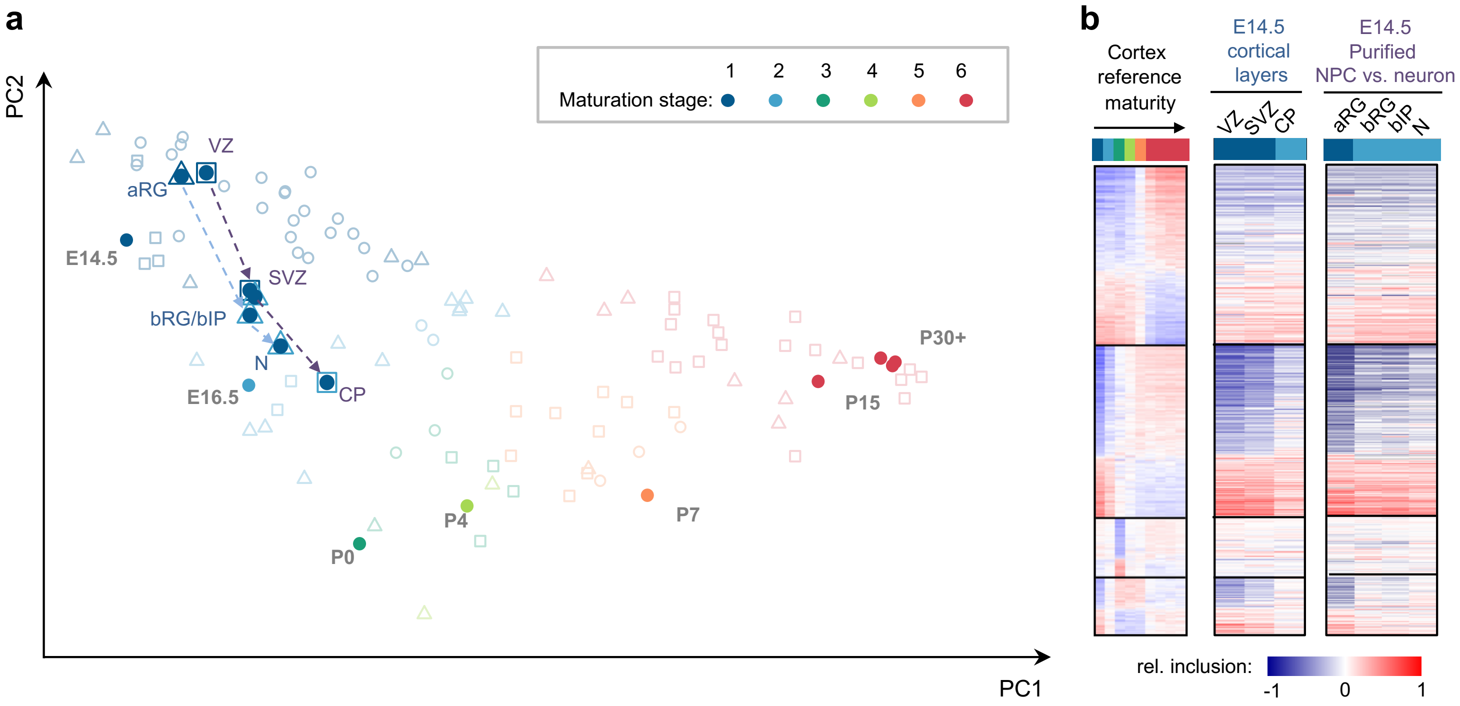

Supplementary Figure 5: Different subpopulations of cells in germininal zones of embryonic cortex show different stages of maturation. **a**, Related to **Fig. 2b** in the main text, but samples from different germinal zones or different cell populations purified from embryonic cortex are labeled. VZ: ventricular zone; SVZ: sub-ventricular zone; CP: cortical plate; aRG: apical radial glial cells; bRG: basal radial glial cells; bIP: basal intermediate progenitor cells; N: neurons. Note that while the correct stage (stage 1) was assigned to samples from the VZ or SVZ-IZ, which are enriched in progenitor cells, a more mature stage (stage 2) was assigned to CP samples enriched in post-mitotic neurons, which is consistent with the pattern of neuron migration and maturation during cortical development; similar classification inaccuracies were made for late-stage radial glial cells and neurons FACS-purified from the embryonic cortex. **b**, Splicing profiles of module exons are shown for samples labelled in panel (a**)**.

UI3 (200 nt)

M1+

#	word	fg	fg+bg	log2FC	pval	FDR	RBP
1	CTCTCT	104	2828	0.74	1.05E-06	4.29E-03	Ptbp
2	CCTTCT	74	1898	0.83	4.92E-06	1.01E-02	Ptbp
3	CCTTTC	72	1849	0.83	6.79E-06	9.28E-03	Ptbp
4	TCTCTC	93	2597	0.70	1.03E-05	1.05E-02	Ptbp
5	CCCTCT	66	1702	0.82	1.79E-05	1.47E-02	Ptbp
6	TTCTCT	107	3193	0.60	3.62E-05	2.47E-02	Ptbp
7	TCCCTC	62	1613	0.81	4.02E-05	2.35E-02	Ptbp
8	GTCTCT	59	1513	0.83	4.10E-05	2.10E-02	
9	TCTCTG	83	2376	0.67	6.65E-05	3.03E-02	
10	GTTTCT	71	1958	0.72	7.16E-05	2.93E-02	
11	CCTCCT	68	1858	0.74	7.66E-05	2.85E-02	
12	TTCCCT	74	2075	0.70	8.53E-05	2.91E-02	
13	TCCCCT	58	1528	0.79	9.62E-05	3.03E-02	
14	CCTCCC	69	1913	0.71	1.04E-04	3.05E-02	
15	TCCCTT	67	1880	0.70	1.80E-04	4.92E-02	
16	TGTCTC	64	1782	0.71	2.01E-04	5.15E-02	
17	CTTCCT	85	2553	0.60	2.55E-04	6.15E-02	
18	TCTCTT	90	2738	0.58	2.60E-04	5.93E-02	
19	CTCCCT	68	1958	0.66	3.27E-04	7.06E-02	
20	TCTGCC	54	1469	0.74	3.48E-04	7.13E-02	

M1-

#	word	fg	fg+bg	log2FC	pval	FDR	RBP
1	CGCATG	12	170	2.38	7.91E-06	3.24E-02	Rbfox
2	CCTGCT	47	1618	1.03	8.50E-06	1.74E-02	Mbnl
3	CTCGCT	15	272	2.00	1.25E-05	1.71E-02	Mbnl
4	TCGCTG	15	278	1.97	1.62E-05	1.66E-02	Mbnl
5	CGCTGC	16	347	1.73	5.74E-05	4.71E-02	Mbnl
6	CCGCAT	9	128	2.37	1.08E-04	7.40E-02	
7	GCACGC	10	158	2.21	1.10E-04	6.45E-02	
8	ATTAAC	21	576	1.37	1.32E-04	6.74E-02	
9	CCCACC	37	1347	0.95	2.12E-04	9.65E-02	
10	CTGTGC	40	1537	0.87	3.55E-04	1.45E-01	
11	GTGTGT	51	2116	0.76	3.72E-04	1.39E-01	
12	TCCCGC	13	292	1.67	3.81E-04	1.30E-01	
13	TGTGTG	61	2716	0.65	6.41E-04	2.02E-01	
14	CCGCCT	12	273	1.65	7.03E-04	2.06E-01	
15	CCGCGG	8	135	2.11	8.09E-04	2.21E-01	
16	GTGCAT	22	714	1.12	8.94E-04	2.29E-01	
17	GCTCCC	25	881	1.00	1.32E-03	3.18E-01	
18	CTGCTG	44	1868	0.72	1.38E-03	3.13E-01	
19	TCCTAA	24	836	1.02	1.40E-03	3.01E-01	
20	CTGCTC	39	1604	0.77	1.43E-03	2.93E-01	

M2+

#	word	fg	fg+bg	log2FC	pval	FDR	RBP
1	TTCTCT	145	3193	1.04	1.11E-14	4.55E-11	Ptbp
2	CTCTCT	132	2828	1.09	2.34E-14	4.79E-11	Ptbp
3	TCTCTT	128	2738	1.09	5.08E-14	6.94E-11	Ptbp
4	TCTCTC	120	2597	1.07	6.41E-13	6.57E-10	Ptbp
5	TCTTTC	111	2534	0.99	1.01E-10	8.26E-08	Ptbp
6	TTTCTC	122	2882	0.94	1.03E-10	7.00E-08	Ptbp
7	CTTTCT	128	3080	0.91	1.16E-10	6.76E-08	Ptbp
8	CTCTGC	84	1845	1.05	3.07E-09	1.57E-06	nSR100
9	TTTCCT	118	3034	0.81	2.21E-08	1.01E-05	Ptbp
10	TCTCCT	91	2171	0.92	3.31E-08	1.36E-05	
11	CTGCCT	83	1950	0.95	6.77E-08	2.52E-05	Mbnl/nSR100
12	TCCTTT	97	2408	0.86	7.63E-08	2.60E-05	Ptbp
13	TTCCTC	81	1894	0.95	7.94E-08	2.50E-05	Ptbp
14	TTCCTT	107	2758	0.81	1.07E-07	3.14E-05	Ptbp
15	CTCTTT	94	2372	0.84	2.43E-07	6.64E-05	Ptbp
16	CTGTCT	93	2356	0.83	3.31E-07	8.47E-05	
17	CCTTTC	77	1849	0.91	4.47E-07	1.08E-04	Ptbp
18	TCTGCT	76	1829	0.91	5.72E-07	1.30E-04	Mbnl/nSR100
19	TCCTCT	83	2089	0.84	1.05E-06	2.27E-04	Ptbp
20	TCTTGT	69	1662	0.91	1.87E-06	3.83E-04	

M2-

#	word	fg	fg+bg	log2FC	pval	FDR	RBP
1	TTTGTT	83	3489	1.08	1.11E-09	4.56E-06	Elavl
2	CATCAT	28	704	1.84	2.36E-08	4.83E-05	Nova
3	TTGTTT	78	3839	0.85	1.66E-06	2.26E-03	Elavl
4	TTTTTT	134	7685	0.62	2.12E-06	2.17E-03	Elavl
5	CCATCA	24	720	1.58	4.79E-06	3.92E-03	Nova
6	TTTTGT	65	3212	0.84	1.28E-05	8.71E-03	Elavl
7	TCCATC	25	852	1.39	2.60E-05	1.52E-02	Nova
8	TTTTTG	50	2345	0.92	3.32E-05	1.70E-02	Elavl
9	TGTTTT	76	4148	0.69	6.66E-05	3.03E-02	Elavl
10	TTGCTT	43	2020	0.91	1.16E-04	4.74E-02	Mbnl
11	TTGCAT	24	909	1.23	1.82E-04	6.78E-02	
12	TGTTTG	45	2196	0.86	1.88E-04	6.41E-02	
13	TCATCT	27	1118	1.10	3.08E-04	9.70E-02	Nova
14	AAATCA	23	888	1.20	3.19E-04	9.34E-02	
15	TATGCA	18	615	1.39	3.36E-04	9.16E-02	
16	GCCATT	20	728	1.29	3.65E-04	9.34E-02	Nova
17	CATGTT	28	1202	1.05	4.24E-04	1.02E-01	
18	TTTTCA	42	2096	0.83	4.66E-04	1.06E-01	
19	GTTTGT	36	1714	0.90	4.97E-04	1.07E-01	
20	TCATGT	26	1117	1.05	6.70E-04	1.37E-01	Nova

DI5 (200 nt)

M1+

#	word	fg	fg+bg	log2FC	pval	FDR	RBP
1	TGCATG	80	1395	1.34	8.41E-13	3.45E-09	Rbfox
2	GTGTGC	67	1182	1.33	8.99E-11	1.84E-07	Celf/nSR100
3	GCATGC	52	826	1.49	3.05E-10	4.16E-07	Rbfox
4	GTGCTT	65	1201	1.26	1.06E-09	1.09E-06	Mbnl
5	TGTGCT	84	1741	1.08	1.14E-09	9.38E-07	Mbnl
6	CCTGCC	77	1612	1.07	8.21E-09	5.61E-06	Mbnl
7	TGCTTC	68	1364	1.13	1.16E-08	6.79E-06	Mbnl
8	CTGGCT	74	1538	1.08	1.18E-08	6.02E-06	
9	GCTTCT	69	1420	1.09	2.44E-08	1.11E-05	
10	TGCTTG	65	1322	1.11	3.95E-08	1.62E-05	Mbnl
11	CCCCCC	57	1103	1.19	5.08E-08	1.89E-05	
12	TGGCTG	73	1585	1.01	8.05E-08	2.75E-05	
13	TGCTGC	66	1396	1.05	1.29E-07	4.06E-05	Mbnl
14	CTGCCT	82	1923	0.89	3.23E-07	9.45E-05	Mbnl
15	GCTTGC	45	834	1.25	3.74E-07	1.02E-04	
16	CTGCTT	74	1684	0.94	3.90E-07	9.97E-05	Mbnl
17	CCATCC	46	916	1.14	1.95E-06	4.71E-04	Nova
18	TGCCTG	70	1639	0.90	2.08E-06	4.74E-04	Mbnl
19	GCATGG	45	905	1.13	3.23E-06	6.95E-04	Rbfox
20	TGTGTG	107	2869	0.69	3.30E-06	6.75E-04	Celf/nSR100

M1-

#	word	fg	fg+bg	log2FC	pval	FDR	RBP
1	CCCCCC	37	1103	1.28	2.29E-06	9.39E-03	
2	CATCGT	11	171	2.26	3.75E-05	7.68E-02	
3	ACAGGT	23	655	1.34	8.73E-05	1.19E-01	
4	ACCCCT	25	775	1.22	1.65E-04	1.69E-01	
5	TGATTG	24	760	1.19	2.98E-04	2.44E-01	
6	CATGTT	28	979	1.04	4.82E-04	3.29E-01	
7	GCTTGC	25	834	1.11	4.83E-04	2.83E-01	
8	TCATCG	8	132	2.17	6.23E-04	3.19E-01	
9	TGTTTG	46	1933	0.77	6.26E-04	2.85E-01	
10	CTCGCT	11	243	1.73	7.88E-04	3.23E-01	
11	TGTGTC	36	1434	0.84	9.24E-04	3.44E-01	
12	CGGTCG	4	31	3.37	9.27E-04	3.16E-01	
13	GCATCG	7	110	2.24	1.01E-03	3.18E-01	
14	AGACGC	8	144	2.04	1.10E-03	3.21E-01	
15	CACCCC	28	1037	0.95	1.14E-03	3.11E-01	
16	GTCTGT	36	1452	0.83	1.14E-03	2.93E-01	
17	CGCTCA	9	185	1.83	1.40E-03	3.37E-01	
18	CTCATT	27	1012	0.93	1.63E-03	3.70E-01	
19	CTGCAC	22	767	1.04	1.75E-03	3.77E-01	
20	TTACTG	25	919	0.96	1.83E-03	3.74E-01	

M2+

#	word	fg	fg+bg	log2FC	pval	FDR	RBP
1	TGCATG	88	1395	1.53	6.62E-17	2.71E-13	Rbfox
2	GCATGC	51	826	1.50	3.69E-10	7.55E-07	Rbfox
3	GCATGT	47	945	1.17	1.00E-06	1.37E-03	Rbfox
4	TGCTCC	48	1017	1.09	3.21E-06	3.29E-03	Mbnl
5	TCCATC	41	819	1.18	4.00E-06	3.28E-03	Nova
6	TGCTGC	60	1396	0.94	4.10E-06	2.80E-03	
7	CTCCAT	46	987	1.07	6.97E-06	4.08E-03	Nova
8	GCATGA	34	662	1.21	1.50E-05	7.66E-03	Rbfox
9	CATGGC	39	829	1.08	2.68E-05	1.22E-02	
10	GCTCCT	48	1115	0.95	3.34E-05	1.37E-02	
11	CCTGTT	48	1145	0.91	6.32E-05	2.35E-02	
12	CCTCCC	61	1580	0.78	7.59E-05	2.59E-02	
13	CCCACC	50	1220	0.87	7.84E-05	2.47E-02	Nova
14	CTGCTT	64	1684	0.76	8.04E-05	2.35E-02	
15	CATGCC	34	724	1.08	8.68E-05	2.37E-02	
16	CTTGTC	41	942	0.96	9.38E-05	2.40E-02	
17	CTGCAT	44	1038	0.92	9.67E-05	2.33E-02	
18	TGCACG	14	184	1.82	9.68E-05	2.20E-02	
19	GTGCAT	37	826	1.01	1.13E-04	2.45E-02	
20	TTCTGC	49	1207	0.86	1.14E-04	2.34E-02	

M2-

#	word	fg	fg+bg	log2FC	pval	FDR	RBP
1	ACTAAC	18	511	1.64	4.24E-05	1.74E-01	QKI
2	ACATCC	17	530	1.50	2.06E-04	4.23E-01	
3	GAGCGT	8	141	2.36	2.64E-04	3.61E-01	
4	ACCATT	18	619	1.35	4.40E-04	4.50E-01	
5	TCCGAG	9	195	2.04	5.12E-04	4.20E-01	
6	ACCGAA	6	88	2.64	5.85E-04	3.99E-01	
7	TTAGAG	22	858	1.17	6.03E-04	3.53E-01	
8	TAACAT	20	760	1.20	7.60E-04	3.89E-01	
9	AACATC	15	492	1.42	7.90E-04	3.60E-01	
10	GCCATC	19	710	1.23	8.35E-04	3.42E-01	
11	CGCACT	7	131	2.27	9.01E-04	3.35E-01	
12	ACGCTC	7	136	2.21	1.12E-03	3.82E-01	
13	TTCTCC	29	1338	0.92	1.32E-03	4.15E-01	
14	TCGCTA	5	71	2.69	1.45E-03	4.23E-01	
15	CGGAGA	8	183	1.96	1.46E-03	3.98E-01	
16	GCTCCT	25	1115	0.97	1.74E-03	4.45E-01	
17	CCATTC	18	702	1.17	1.79E-03	4.31E-01	
18	AAATTC	20	831	1.07	2.16E-03	4.91E-01	
19	CCGAGC	8	195	1.87	2.17E-03	4.67E-01	
20	CCAGCT	26	1201	0.92	2.29E-03	4.69E-01	

Supplementary Figure 6: ***De novo*** **motif analysis on WGCNA module exons.** Core exons in modules M1 and M2 in each direction were compared with all mouse cassette exons to evaluate the enrichment of each hexamer in the upstream and downstream intronic sequences (200 nt on each side). Only the top 20 words are shown and hexamers that resemble consensus binding sites of known RBPs are indicated. Motif enrichment in the alternative exon is also evaluated but no significant hexamers were found.

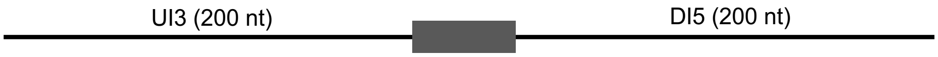

Motif	fg	log2FC	pval		fg	log2FC	pval
TCTY (Ptbp)	**1219**	**0.30**	**7.40E-13**		891	0.11	1.03E-02
YCAY (Nova)	1085	0.10	9.46E-03	M1+	1097	0.11	5.49E-03
TGCATG (Rbfox)	33	0.36	9.50E-02		**80**	**1.34**	**8.41E-13**
YGCY (Mbnl)	876	0.06	1.17E-01		**1271**	**0.59**	**3.72E-42**
TCTY (Ptbp)	689	0.11	2.70E-02		532	0.10	6.76E-02
YCAY (Nova)	704	0.11	2.08E-02	M1-	690	0.17	1.22E-03
TGCATG (Rbfox)	27	0.71	1.15E-02		31	0.66	1.08E-02
YGCY (Mbnl)	**733**	**0.44**	**1.94E-15**		587	0.19	1.09E-03
TCTY (Ptbp)	**1392**	**0.48**	**1.64E-32**		864	0.11	1.72E-02
YCAY (Nova)	1020	0.00	5.33E-01	M2+	**1233**	**0.32**	**2.14E-14**
TGCATG (Rbfox)	34	0.39	7.62E-02		**88**	**1.53**	**6.62E-17**
YGCY (Mbnl)	1079	0.35	1.13E-14		1069	0.37	8.21E-16
TCTY (Ptbp)	504	-0.01	5.67E-01		437	0.10	8.45E-02
YCAY (Nova)	**664**	**0.37**	**1.63E-10**	M2-	534	0.08	9.17E-02
TGCATG (Rbfox)	**25**	**0.94**	**2.29E-03**		17	0.07	4.52E-01
YGCY (Mbnl)	479	0.16	1.01E-02		453	0.10	7.98E-02

Supplementary Figure 7: Consensus motif analysis on WGCNA module exons. Core exons in modules M1 and M2 in each direction were compared with all mouse cassette exons to evaluate the enrichment of the consensus motif for Nova, Rbfox, Mbnl and Ptbp in the upstream and downstream intronic sequences (200 nt on each side).

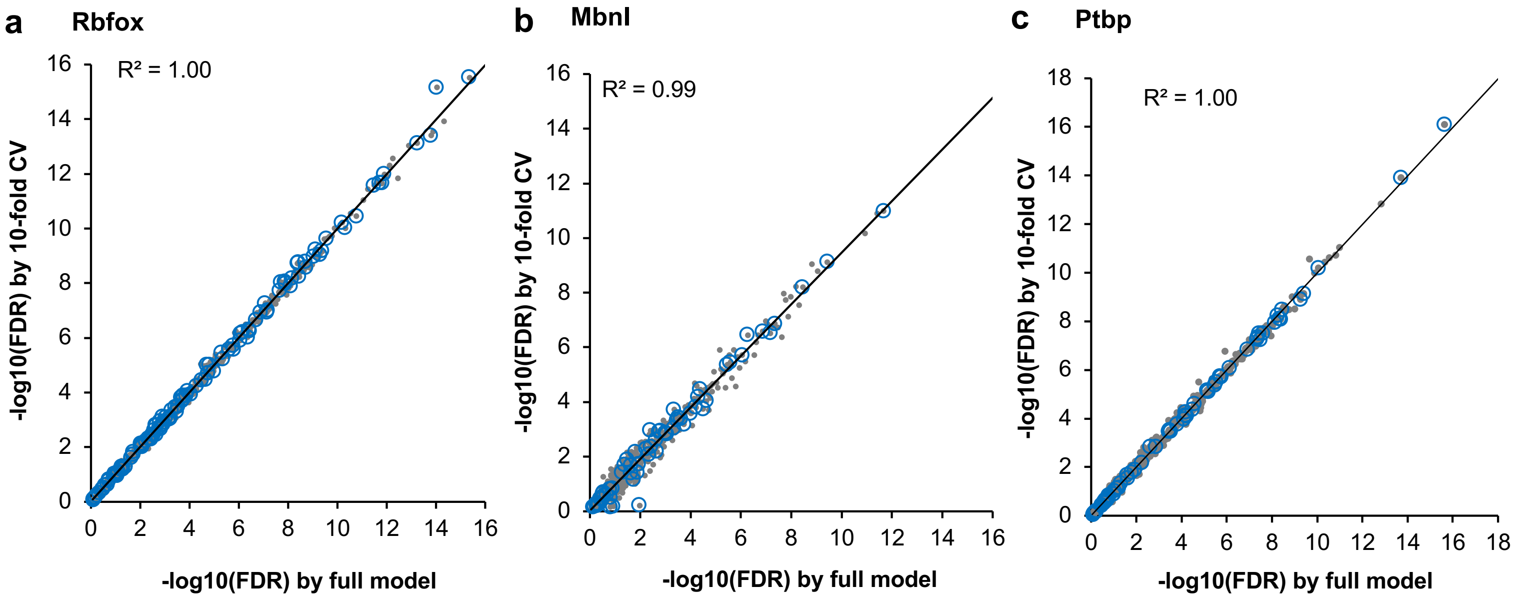

Supplementary Figure 8: 10-fold cross validation of Bayesian network analysis to predict RBP target exons. a, Rbfox. **b**, Mbnl. **c**, Ptbp. In each panel, exons used for model training and cross validation are shown. Previously validated exons are highlighted in blue.

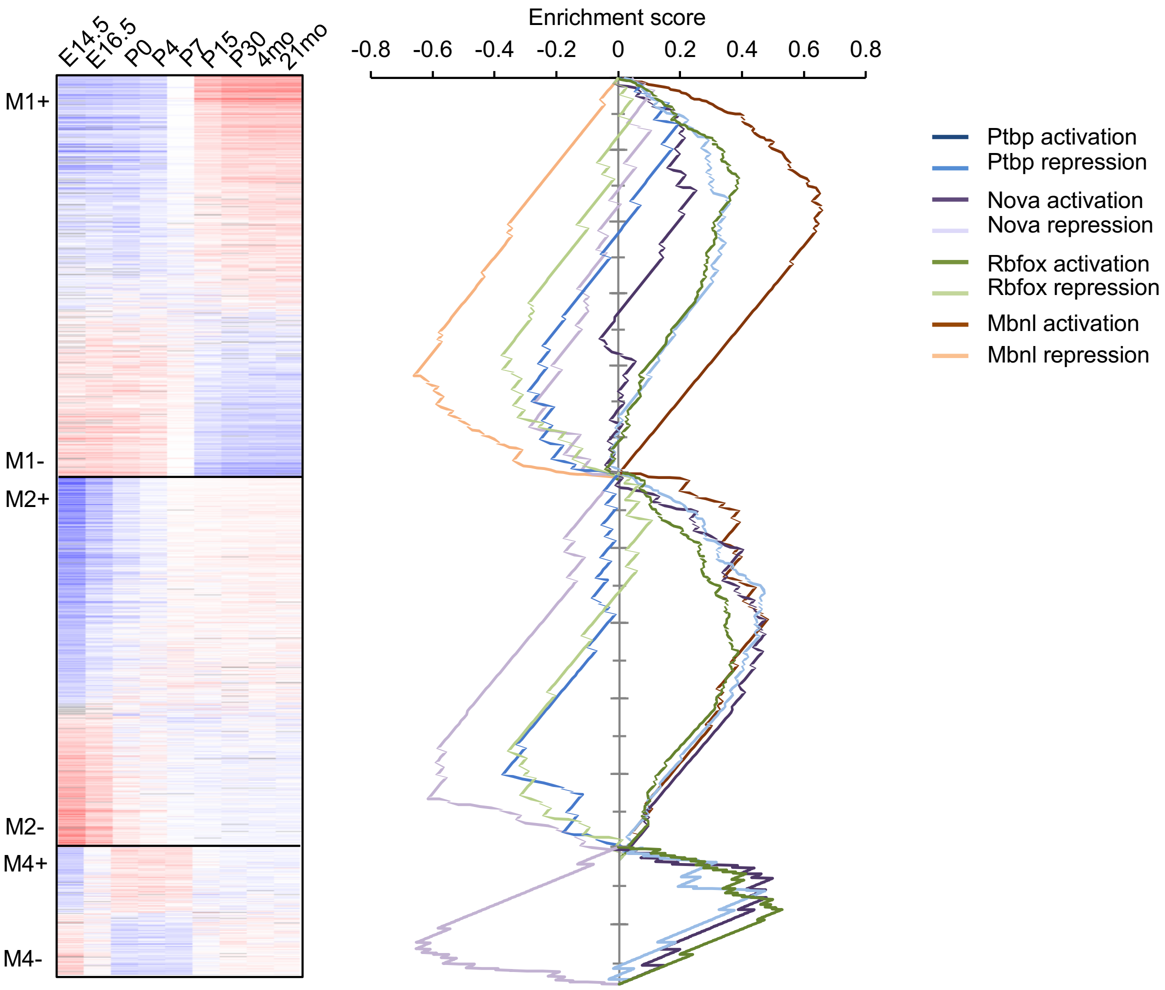

Supplementary Figure 9: Gene Set Enrichment Analysis (GSEA) of RBP targets in WGCNA module exons. GSEA was performed for exons in modules M1, M2 and M4 separately. Each gene set is defined by the group of target exons activated or repressed by specific RBPs.

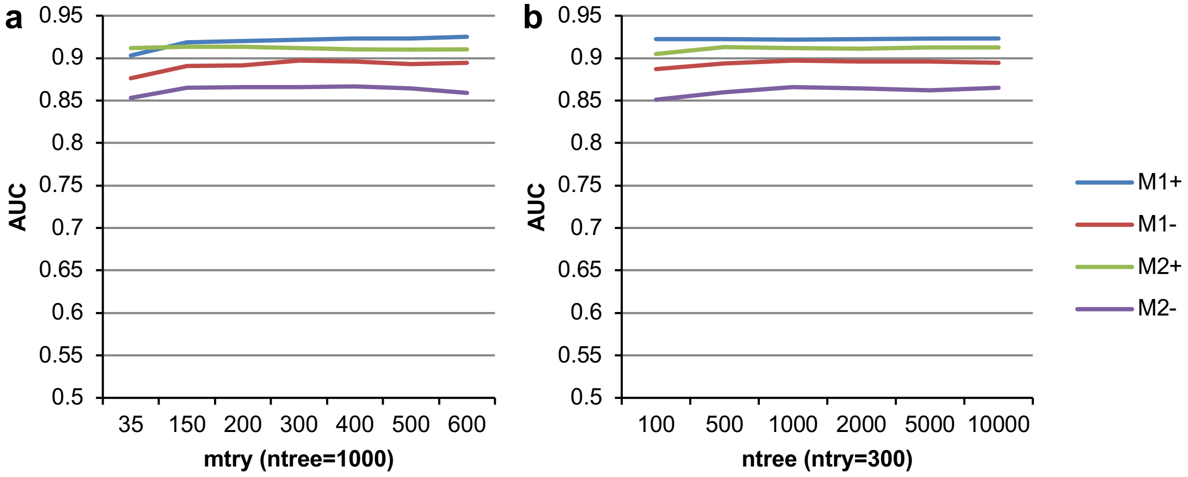

Supplementary Figure 10: Performance of random forest in predicting module exons using different parameters. **a**, Performance vs. number of variables per tree in the forest (mtry). **b**, Performance vs. number of trees in the forest (ntree).

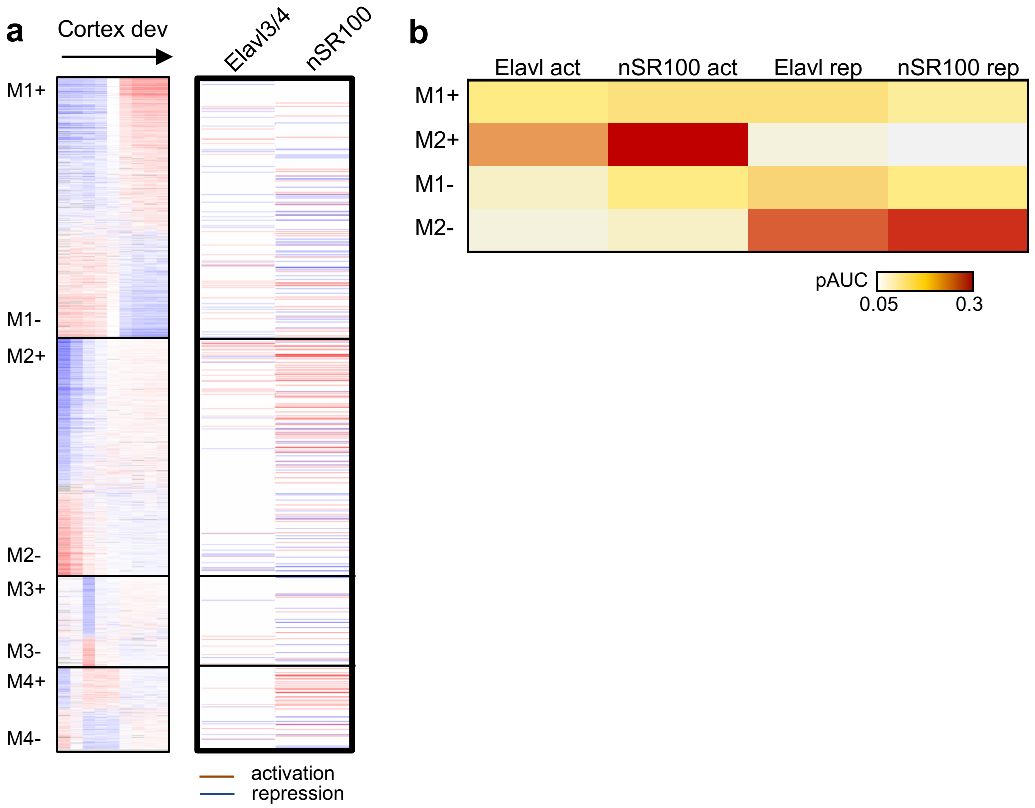

Supplementary Figure 11: Additional neuronal RBPs contributing to early splicing switches. **a**, Activation or repression of module exons by Elavl3/4 and nSR100. Elavl3/4-dependent exons were identified by comparison of WT and Elavl3/4 dKO mouse cortices using exon-junction microarrays (Ince-Dunn et al.; $|\Delta \text{IRank}| \geq 6.5$). nSR100 (SRRM4)-dependent exons were identified by comparison of WT and nSR100 KO mouse hippocampi using RNA-seq ($|\Delta\Psi| \geq 0.1$, Benjamini FDR$\leq$0.05). **b**, Similar to Fig. 4j in the main text. Prediction performance of exon module membership based on regulation by each RBP family. Activation or repression by each RBP as determined from exon-junction microarrays or RNA-seq was used to predict early and late splicing switches, as well as the direction of switches. The performance is measured by partial area under curve (pAUC) of the receiver operating characteristic (ROC) plot with a cutoff at false positive rate (FPR)$\leq$0.1.

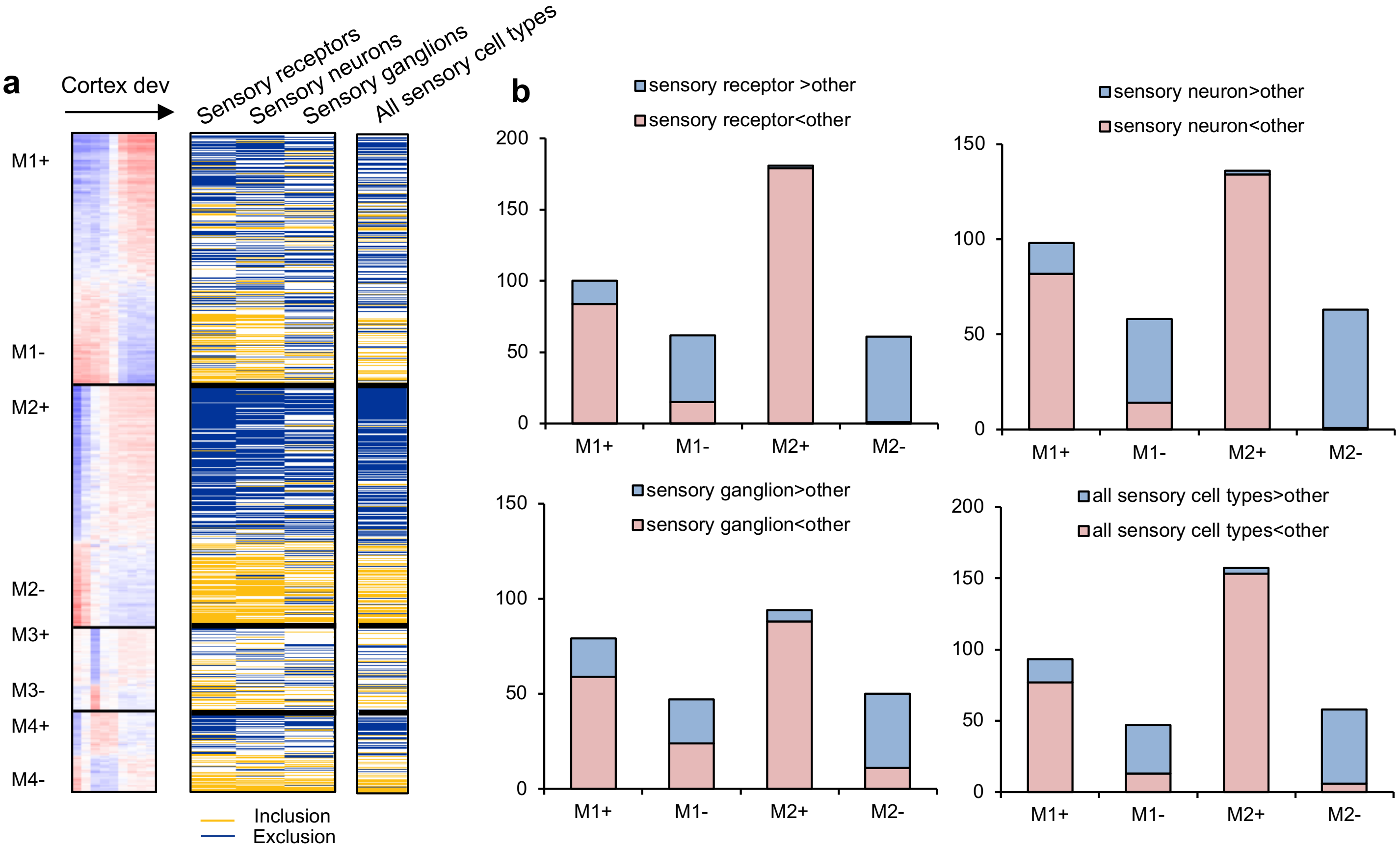

Supplementary Figure 12: Differential splicing analysis of peripheral and sensory neurons compared to mature CNS neurons. **a**, Heatmap showing exons with statistically significant inclusion (yellow) and exclusion (blue) in each type of peripheral and sensory cells (sensory receptors, sensory neurons and sensory ganglion neurons) compared to the mature CNS neurons. **b**, Overlap between core module exons and exons showing differential splicing in each type of peripheral sensory cells compared to mature CNS neurons. Exons with increased or decreased inclusion in each module and direction are shown separately.

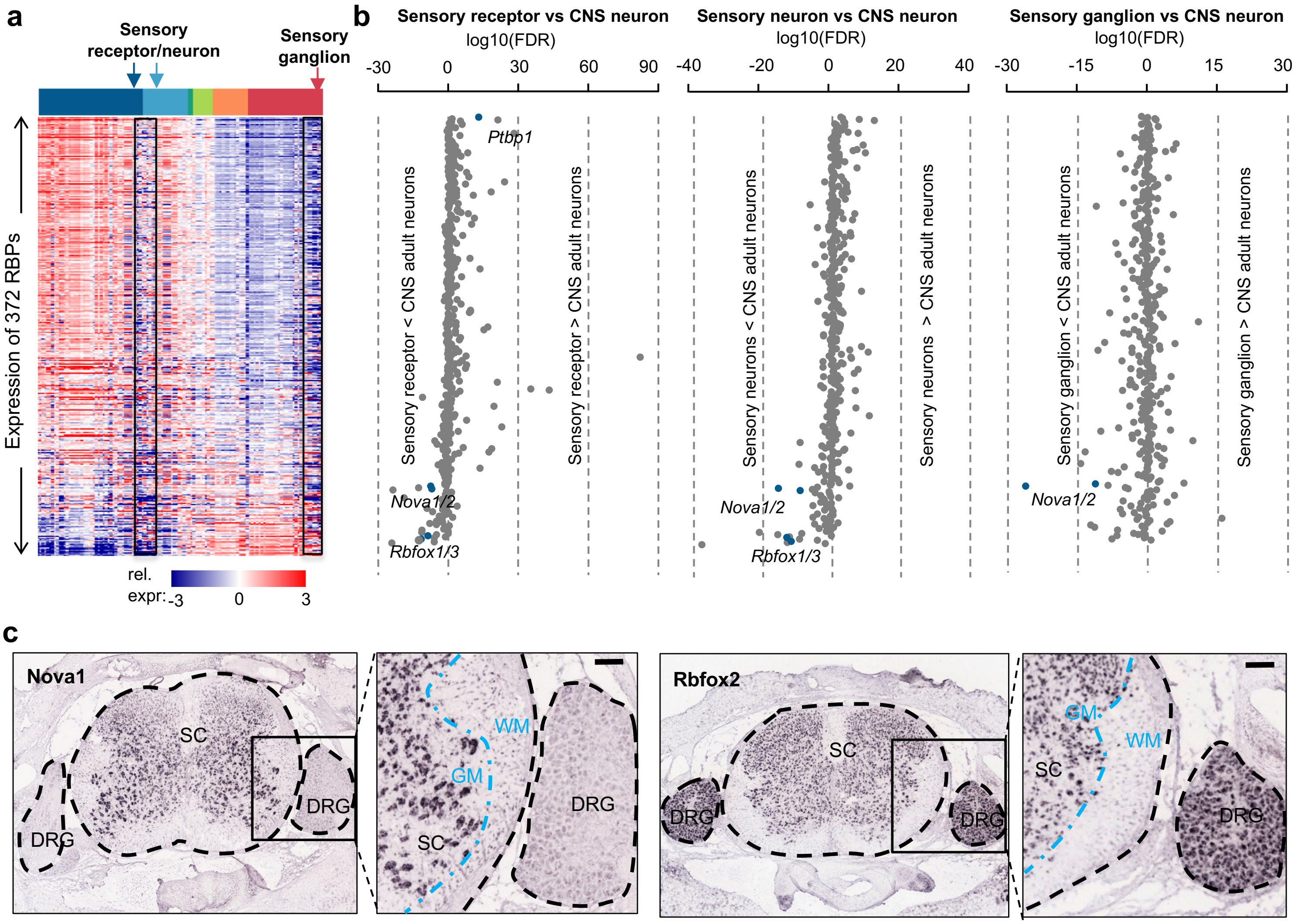

Supplementary Figure 13: Only specific RBPs show distinct expression in peripheral and sensory neurons compared to mature CNS neurons. **a**, Expression of RBPs (log2 transformed, median centered RPKM values) across different tissue or neuronal samples was used in the analysis. This analysis included all RBPs compiled in RBPDB (http://rbpdb.ccbr.utoronto.ca). The same list of 346 samples used to predict maturation stages (Supplementary Dataset 1) was analyzed. Samples were ordered by the predicted maturation stage, and RBPs were ordered by the correlation of their expression with the predicted sample maturation stages. Sensory cell types are highlighted. The data matrix used to generate this heatmap is available in **Supplementary Dataset 13**. **b**, Differential expression analysis of RBPs in each cell type from the peripheral and sensory system compared to mature CNS neurons purified from adult mouse brains. The Benjamini FDR (in log2 scale) with sign indicating the direction of expression difference is shown. RBPs are shown in the same order as in (a). A subset of RBPs are highlighted. **c**, Nova1 (top) and Rbfox2 (bottom) expression in P4 mouse spinal cord using in situ hybridization. Data were obtained from Allen Brain Atlas (http://mousespinal.brain-map.org). In the spinal cord, gray matter and white matter are indicated due to neuron-specifix or enriched expression of Nova and Rbfox2. Probes for Nova2 and Rbfox1/3 are not available. SC, spinal cord; DRG, dorsal root ganglion; WM, white matter; GM, gray matter. Scale bar: 100 μm.

Supplementary References

1. Kosik, K.S., Orecchio, L.D., Bakalis, S. & Neve, R.L. Developmentally regulated expression of specific tau sequences. *Neuron* **2**, 1389-97 (1989).
2. Tasic, B. *et al.* Adult mouse cortical cell taxonomy revealed by single cell transcriptomics. *Nat Neurosci* **19**, 335-46 (2016).
3. St-Jeannet, J.P. & Moody, S.A. Establishing the pre-placodal region and breaking it into placodes with distinct identities. *Developmental Biology* **389**, 13-27 (2014).
4. Graziadei, P.P. & Monti Graziadei, G.A. Neurogenesis and plasticity of the olfactory sensory neurons. *Ann N Y Acad Sci* **457**, 127-42 (1985).
5. Gans, C. & Northcutt, R.G. Neural crest and the origin of vertebrates: a new head. *Science* **220**, 268-73 (1983).
6. Lopes, D.M., Denk, F. & McMahon, S.B. The molecular fingerprint of dorsal root and trigeminal ganglion neurons. *Front Mol Neurosci* **10**, 304 (2017).
7. Steventon, B., Mayor, R. & Streit, A. Neural crest and placode interaction during the development of the cranial sensory system. *Dev Biol* **389**, 28-38 (2014).
8. Lamb, T.D., Collin, S.P. & Pugh, E.N., Jr. Evolution of the vertebrate eye: opsins, photoreceptors, retina and eye cup. *Nat Rev Neurosci* **8**, 960-76 (2007).
9. Fuhrmann, S. Eye morphogenesis and patterning of the optic vesicle. *Curr Top Dev Biol* **93**, 61-84 (2010).
