# Supplementary material for: Precise temporal regulation of alternative splicing during neural development
Source: Nat Commun. 2018 Jun 6;9:2189. doi: 10.1038/s41467-018-04559-0 (PMC5989265; doi:10.1038/s41467-018-04559-0)
Supplement: Supplementary file 2 — Description of Additional Supplementary Files [file 41467_2018_4559_MOESM2_ESM.pdf]

**Description of Additional Supplementary Files**

File Name: Supplementary Data 1
Description: Summary of RNA-Seq data analyzed in this study.

File Name: Supplementary Data 2
Description: Oligonucleotide sequences used to in this study.

File Name: Supplementary Data 3
Description: Developmentally regulated exons compiled from literature and the primers used for RT-PCR validation.

File Name: Supplementary Data 4
Description: Summary of AS events with significant developmental splicing changes.

File Name: Supplementary Data 5
Description: WGCNA module assignment of developmentally regulated exons.

File Name: Supplementary Data 6
Description: Summary of sliding window GO analysis (Benjamini FDR<0.005).

File Name: Supplementary Data 7
Description: Summary of module GO analysis (Benjamini FDR<0.05).

File Name: Supplementary Data 8
Description: Summary of neuronal maturation stages predicted from splicing profiles.

File Name: Supplementary Data 9
Description: Summary of direct RBP target exons predicted by Bayesian network analysis.

File Name: Supplementary Data 10
Description: Sensitivity and specificity of Bayesian network analysis.

File Name: Supplementary Data 11
Description: Top 10 words used to score additional RBP motif sites. The words were ranked based on their binding affinity to the corresponding RBPs as determined by RNACompete.

File Name: Supplementary Data 12
Description: List of features for random forest analysis and their importance.

File Name: Supplementary Data 13
Description: RPKM values of 372 RBPs in all samples used for maturation predication.
